# MicroRNA-143 inhibits cell growth by targeting ERK5 and MAP3K7 in breast cancer

**DOI:** 10.1590/1414-431X20175891

**Published:** 2017-07-17

**Authors:** L.L. Zhou, J.L. Dong, G. Huang, Z.L. Sun, J. Wu

**Affiliations:** 1 Department of Cardiology, Huaihe Hospital of Henan University, Kaifeng, China; 2 Department of Pathology, Huaihe Hospital of Henan University, Kaifeng, China

**Keywords:** Extracellular signal-regulated kinase 5, Mitogen-activated protein 3 kinase 7, miRNA-143, Breast cancer, Cyclin D1

## Abstract

This study aimed to investigate the function and mechanism of microRNA-143 (miR-143) in the occurrence and development of breast cancer (BC). A total of 30 BC tissues, 30 corresponding noncancerous tissues, and 10 normal control (NC) breast tissues were obtained to detect the levels of miR-143, extracellular signal-regulated kinase 5 (ERK5) and mitogen-activated protein 3 kinase 7 (MAP3K7) using RT-qPCR, western blotting or immunohistochemistry. The correlation of miR-143 with ERK5 or MAP3K7 was evaluated using Pearson correlation analysis. MCF-7 cells were transiently transfected with miR-143 mimic, miR-143 inhibitor, miR-143 mimic/inhibitor + si-ERK5, si-MAP3K7 or si-cyclin D1. Then, cell growth was evaluated by MTT assay and the expressions of phospho-ERK5 (p-ERK5), ERK5, p-MAP3K7, MAP3K7 and cyclin D1 were detected by western blotting. Results showed that, compared with noncancerous tissues or NC breast tissues, miR-143 level was decreased, while p-ERK5, ERK5, p-MAP3K7 and MAP3K7 expressions were increased in BC tissues (all P<0.01). The miR-143 level was negatively correlated with the mRNA level of ERK5 or MAP3K7 (*r*=-4.231 or *r*=-4.280, P<0.01). In addition, up-regulated miR-143 significantly decreased the expressions of p-ERK5, ERK5, p-MAP3K7, MAP3K7 and cyclin D1 (all P<0.01), as well as cell viability in MCF-7 cells (all P<0.05) while the effect of down-regulated miR-143 was the opposite. In conclusion, both ERK5 and MAP3K7 may be the target genes of miR-143. Increased expression of miR-143 can inhibit cell growth, which may be associated with ERK5 and MAP3K7 expressions in BC.

## Introduction

Breast cancer (BC) is a prevalent malignant disease in women worldwide and is the third leading cause of cancer-related death (1). Approximately 1.6 million newly diagnosed BC and 1.2 million deaths caused by BC are reported each year in China (2). A previous study has shown a 90% 5-year survival rate in early stage BC, while only a 23% rate was found in advanced stage BC (2). Although much effort has been made to develop effective diagnostic and treatment methods, clinical benefits and survival are still unsatisfactory due to delayed diagnosis and endocrine resistance (3). Therefore, it is imperative to investigate the underlying pathogenesis and search for effective diagnostic and therapeutic targets in BC.

MicroRNAs (miRNAs), small non-coding RNAs, play significant regulatory roles in oncogenesis or as antioncogenes through mediating target gene expressions and pathways related to cancer (4). Evidence suggests that the functions of miRNAs are involved in immune response, inflammation reaction, infection as well as cell metabolism, growth and migration (5). Accumulating studies have focused on the potential roles of miRNAs in various types of cancers, such as colorectal cancer (6), hepatocellular carcinoma (7) and breast cancer (8).

MiR-143, a miRNA located in chromosome 5q33, is reported to be down-regulated in non-small cell lung cancer (9), colorectal cancer (10), prostate cancer (11) and BC (12). MiR-143 has been proved to have an anti-cancer effect by targeting multiple genes related to cell proliferation, apoptosis and migration, such as Bcl-2 (13), MYO6 (14), ELK1 (15), and ERK5 (16). Extracellular signal-regulated kinase 5 (ERK5), a mitogen-activated protein kinase (MAPK), is commonly reported as a target gene of miR-143 (17,18). Previous study has suggested that the activation of ERK5 can be induced by a series of cellular mitogens and stresses, and is associated with cell proliferation and differentiation (17). Importantly, ERK5 is reported to target some well-known regulators related to cell proliferation, including nuclear factor (NF)-κB (NF-κB), c-Myc and cyclin D1 (17). In addition, mitogen-activated protein 3 kinase 7 (MAP3K7), also named transforming growth factor (TGF-β)-activated kinase-1, is a serine/threonine kinase (19). It plays an important role in cell proliferation by regulating various cellular pathways, including TGF-β signal transduction, NF-κB, JNK, and p38 signaling pathway (20). However, few studies evaluate the relationship of miR-143 and MAP3K7 in BC, and the mechanism and function of miR-143 in the occurrence and development of BC are still not fully understood.

In the present study, we compared the levels of miR-143, ERK5, and MAP3K7 in BC tissues, noncancerous tissues and normal breast tissues. Next, we up- or down-regulated the miR-143 level in human breast cancer cell line MCF-7, aiming to explore the effects of miR-143 on cell growth and the expressions of ERK5 and MAP3K7 in BC.

## Material and Methods

### Tissue samples and cell line

A total of 30 primary BC tissues and 30 corresponding noncancerous tissues (>2 cm from BC tissues) were collected from primary BC patients who underwent tumors surgical resection. Ten normal control (NC) breast tissues were collected from age- and gender-matched patients who underwent mammoplasty. This study obtained the approval of the Ethics Committee of the Huaihe Hospital of Henan University and written informed consent from each patient. MCF-7 cells were purchased from Shanghai Cell Bank of Chinese Academy of Sciences (China). The cells were cultured in RPMI-1640 medium (Gibco, USA) containing 10% fetal bovine serum (FBS; Gibco).

### Cell treatment

MCF-7 cells were seeded onto 60-mm dishes and cultured for 24 h. Cells were then transiently transfected with 1 nm of miR-143 mimic or miR-143 inhibitor (Qiagen, USA) using HiPerFect Transfection Reagent (Qiagen). Meanwhile, mimic control and inhibitor control served as negative control, respectively. To investigate the relationship of miR-143 and ERK5, MAP3K7 or cyclin D1, cells were co-transfected with miR-143 mimic/inhibitor and small interfering RNA targeting (si)-ERK5, si-MAP3K7 or si-cyclin D1 (100 nm, Qiagen). After treatment for 24 h, cells with various treatments were subjected to the following experiments.

### Real-time quantitative PCR (RT-qPCR)

Total RNA from cells and tissues was extracted using Trizol reagent (Invitrogen, USA), and miRNAs were obtained using miRneasy Kit (Qiagen). Then, the levels of miR-143, ERK5 and MAP3K7 were detected using SYBR¯ Premix Ex Taq™ (TaKaRa, Japan). The PCR primers for miR-143 and U6 were commercially obtained from Applied Biosystems (USA). ERK5 sense primer was 5′-CTGGCTGTCCAGATGTGAA-3′ and antisense primer 5′-ATGGCACCATCTTTCTTTGG-3′. MAP3K7 sense primer was 5′- ACTCACTTGATGCGGT-3′ and antisense primer 5′-CGGCGATCCTAGCTTC-3′. Glyceraldehyde-3-phosphate dehydrogenase (GAPDH) sense primer was 5′-GACGGCCGCATCTTCTTGT-3′ and antisense primer 5′-CACACCGACCTTCACCATTTT-3′. PCR amplification was performed based on the following program: 95°C for 3 min, 40 cycles of 95°C for 10 s, and 55°C for 30 s. The relative quantification was calculated by comparative threshold (Ct) cycle method (2^-ΔΔCt^) and normalized with U6 SnRNA or GAPDH mRNA.

### Immunohistochemistry (IHC) for ERK5 and MAP3K7

BC tissues, noncancerous tissues and NC breast tissues were fixed in 4% paraformaldehyde. Paraffin sections (4-μm thick) of tissues were dewaxed and dehydrated in xylene and gradient ethanol, respectively. Heated-citrate buffer solution, pH 6.0, was used for antigen retrieval. Endogenous peroxidase activity was carried out using 3% hydrogen peroxide solution for 10 min at room temperature. Next, sections were blocked with 1% serum, and then incubated with rabbit anti-human ERK5, phospho-ERK5 (p-ERK5), MAP3K7 or phospho-MAP3K7 (p-MAP3K7) polyclonal antibody (1:50, Santa Cruz, USA) at 4°C overnight. Phosphate-buffered saline (PBS) was used as the primary antibody in the negative controls. After washing with PBS, sections were incubated with secondary antibody for 45 min, and then stained with diaminobenzidine. Lastly, all sections were re-stained with hematoxylin, dehydrated and sealed. Sections were observed under a light microscope (Nikon, Japan) and positive staining was defined as brown particles in the cytomembrane or cytoplasm. The quantification of positive cells was analyzed by ImagePro Plus software (Cybernetics, Inc., USA).

### MTT assay

A total of 1×10^4^ MCF-7 cells were cultured in 96-well plate for 24 h. After treatment with above transfections for 24, 48, 72, or 96 h, MTT (10 μL, 5 mg/mL, Sigma, USA) was added into each well and maintained for 4 h at 37°C. Subsequently, dimethyl sulfoxide (100 μL, DMSO, Sigma) was added to dissolve the formazan crystals at room temperature. The absorbance value at 490 nm was obtained using a microplate reader (Molecular Devices, USA).

### Western blotting

Protein from cells was extracted using RIPA lysis buffer (Beyotime Institute of Biotechnology, China) and concentration was measured using the BCA Protein Quantitative Assay (Beyotime Institute of Biotechnology). A total of 50 μg protein sample (per lane) was separated on SDS-PAGE gel, blotted onto PVDF membranes, and blocked in 5% nonfat milk for 1 h. The membranes were probed with mouse anti-phospho-ERK5 (p-ERK5), ERK5, p-MAP3K7, MAP3K7 and cyclin D1 polyclonal antibodies (1:500, Santa Cruz) and mouse anti-GAPDH monoclonal antibody (1:2000, Sigma) overnight at 4°C, respectively. After washing three times with PBS, the membranes were incubated with appropriate IgG (H+L)-HRP (1:5000, Santa Cruz) second antibody for 2 h at room temperature. Ultimately, the proteins were detected with enhanced chemiluminescence (Millipore, USA).

### Statistical analysis

Statistical analysis was carried out using the SPSS 19.0 software (SPSS Inc., USA). Data are reported as means±SD and were analyzed by one-way analysis of variance. Pearson correlation analysis was used to evaluate the correlation of miR-143 with ERK5 or MAP3K7. P<0.05 was considered to be significant.

## Results

### Relationship of miR-143 and ERK5 or MAP3K7 in BC tissues

**Figure 1.**
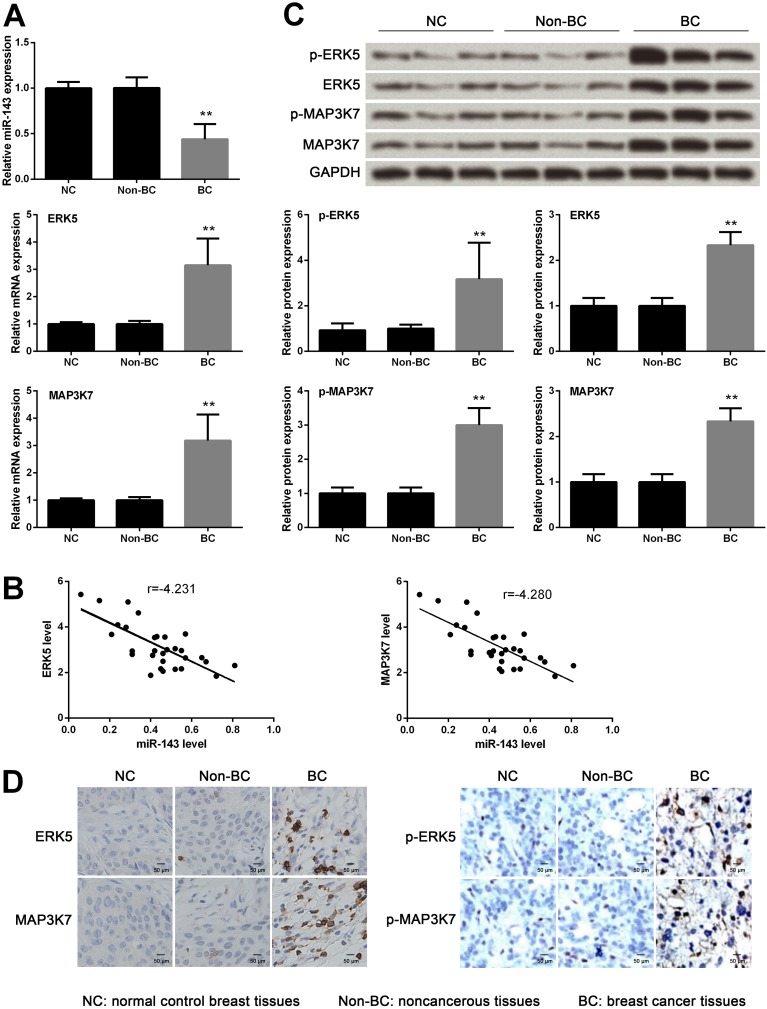
*A*, mRNA levels of miR-143, ERK5 and MAP3K7 in breast cancer (BC) tissues, noncancerous tissues and normal control (NC) breast tissues using RT-qPCR. *B*, Correlation of miR-143 with ERK5 and MAP3K7 mRNA levels in BC tissues by Pearson correlation analysis. *C*, Expression and phosphorylation levels of ERK5 and MAP3K7 in BC tissues, noncancerous tissues and NC breast tissues by western blotting. *D*, Expression and phosphorylation of ERK5 and MAP3K7 by immunohistochemistry. ERK5: extracellular signal-regulated kinase 5; MAP3K7: mitogen-activated protein 3 kinase 7. Data are reported as means±SD of 30 samples. Each analysis was repeated three times. **P<0.01 compared with noncancerous tissues or NC breast tissues (ANOVA).

RT-qPCR analysis revealed that the miR-143 level was decreased in BC tissues compared with noncancerous tissues or NC breast tissues (P<0.01). The mRNA levels of ERK5 and MAP3K7 were increased in BC tissues compared with noncancerous tissues or NC breast tissues (P<0.01, Figure 1A). Moreover, Pearson correlation analysis showed that the miR-143 level was negatively correlated with the mRNA level of ERK5 or MAP3K7 (*r*=-4.231 or *r*=-4.280, P<0.01, Figure 1B). In addition, the protein expressions of ERK5, p-ERK5, MAP3K7 and p-MAP3K7 were all significantly increased in BC tissues compared with noncancerous tissues or NC breast tissues (P<0.01, Figure 1C). IHC results also showed the higher positive expressions (brown particles) of ERK5, p-ERK5, MAP3K7 and p-MAP3K7 in BC tissues than those in noncancerous tissues or NC breast tissues (Figure 1D).

### Effect of ectopic miR-143 level on the expression and phosphorylation of ERK5 and MAP3K7 in MCF-7 cells

**Figure 2.**
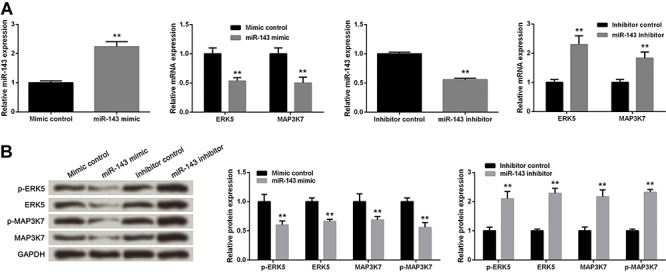
Up-regulated miR-143 decreased expression and phosphorylation of ERK5and MAP3K7 in MCF-7 cells. Cells were transfected with mimic control, miR-143 mimic, inhibitor control or miR-143 inhibitor. *A*, miR-143 levels and mRNA levels of ERK5 and MAP3K7 using RT-qPCR. *B*, Expression and phosphorylation levels of ERK5 and MAP3K7 using western blot analysis. ERK5: extracellular signal-regulated kinase 5; MAP3K7: mitogen-activated protein 3 kinase 7. Data are reported as means±SD of three independent experiments. **P<0.01 compared with control (ANOVA).

RT-qPCR analysis found that the miR-143 level was significantly increased in cells with miR-143 mimic compared with cells with mimic control (P<0.01), and the mRNA levels of ERK5 and MAP3K7 in cells with miR-143 mimic were remarkably decreased compared with cells with mimic control (P<0.01, Figure 2A), which was consistent with the protein expressions detected by western blotting results (P<0.01). The protein expressions of p-ERK5 and p-MAP3K7 were both significantly decreased in cells with miR-143 mimic compared with cells with mimic control (P<0.01, Figure 2B). In addition, the transfection of miR-143 inhibitor in MCF-7 cells obviously inhibited the miR-143 level compared with the transfection of inhibitor control (P<0.01, Figure 2A). The down-regulation of miR-143 markedly elevated the mRNA and protein levels of ERK5 and MAP3K7 as well as protein expressions of p-ERK5 and p-MAP3K7 compared with cells with inhibitor control (P<0.01, Figure 2A and B).

### Effect of ectopic miR-143, ERK5 and MAP3K7 levels on cell growth in MCF-7 cells

**Figure 3.**
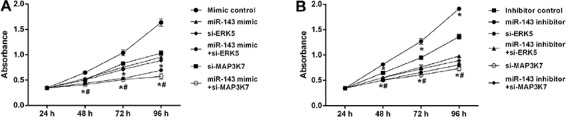
MTT cell viability assay for *A*, cells transfected with mimic control, si-ERK5, si-MAP3K7, miR-143 mimic alone or accompanied by si-ERK5 or si-MAP3K7 and *B*, cells transfected with inhibitor control, si-ERK5, si-MAP3K7, miR-143 inhibitor alone or accompanied by si-ERK5 or si-MAP3K7. ERK5: extracellular signal-regulated kinase 5; MAP3K7: mitogen-activated protein 3 kinase 7; si: small interfering RNA targeting. Data are reported as means±SD of three independent experiments. *P<0.05 compared with mimic or inhibitor control, ^#^P<0.05 compared with miR-143 mimic or inhibitor (ANOVA).

MTT assay showed that compared with cells with mimic control, cell viability was significantly reduced in cells with miR-143 mimic, si-ERK5 or si-MAP3K7 at 48, 72, and 96 h (all P<0.05). When cells were co-treated with miR-143 mimic and si-ERK5 or si-MAP3K7 for 48, 72, and 96 h, cell viability was markedly lower than those in cells with other treatments, including mimic control, miR-143 mimic, si-ERK5 and si-MAP3K7 (all P<0.05, Figure 3A). Consistently, down-regulated miR-143 significantly promoted cell viability compared with cells with inhibitor control at 48, 72, and 96 h (all P<0.05). In addition, cells with co-transfection of miR-143 inhibitor and si-ERK5 or si-MAP3K7 showed lower cell viability than cells with miR-143 inhibitor or inhibitor control at 48, 72, and 96 h (all P<0.05, Figure 3B).

### Effect of miR-143 and cyclin D1 on cell growth in MCF-7 cells

**Figure 4.**
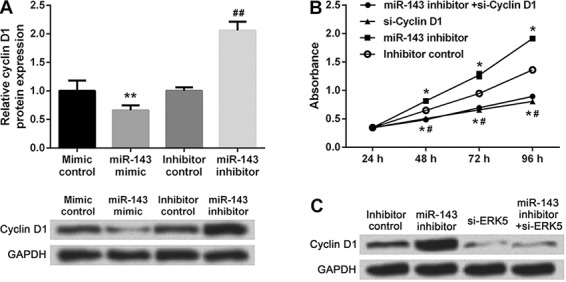
*A*, Cyclin D1 expression in cells transfected with mimic control, miR-143 mimic, inhibitor control or miR-143 inhibitor, detected by western blotting. **P<0.01 compared with mimic control, ^##^P<0.01 compared with inhibitor control (ANOVA). *B*, Cell viability in cells transfected with inhibitor control, si-cyclin D1, miR-143 inhibitor alone or accompanied by si-cyclin D1, detected by MTT assay. *P<0.05 compared with inhibitor control, ^#^P<0.05 compared with miR-143 inhibitor (ANOVA). *C*, Cyclin D1 expression in cells transfected with inhibitor control, si-ERK5, miR-143 inhibitor alone or accompanied by si-ERK5. ERK5: extracellular signal-regulated kinase 5; si: small interfering RNA targeting. Data are reported as means±SD of three independent experiments. Each experiment was repeated three times.

Western blotting results showed that the expression of cyclin D1 in cells with miR-143 mimic were remarkably decreased compared with cells with mimic control (P<0.01), and the down-regulated miR-143 markedly elevated the protein level of cyclin D1 compared with cells with inhibitor control (P<0.01, Figure 4A). When cells were co-transfected with miR-143 inhibitor and si-cyclin D1, cell viability was significantly reduced compared with cells with miR-143 inhibitor or inhibitor control at 48, 72, and 96 h (all P<0.05, Figure 4B). Besides, the increase of cyclin D1 expression induced by miR-143 down-regulation was reversed by si-ERK5 (Figure 4C).

## Discussion

miRNAs have been reported to be novel biomarkers for BC (21). In the present study, the level of miR-143 was reduced, while the expression and phosphorylation of ERK5 and MAP3K7 were increased in BC tissues compared with noncancerous tissues or NC breast tissues. The miR-143 level was negatively correlated with the ERK5 or MAP3K7 expression. In MCF-7 cells, up-regulated miR-143 significantly decreased the expression and phosphorylation levels of ERK5 and MAP3K7, and up-regulated miR-143 and down-regulated ERK5 or MAP3K7 markedly reduced cell viability. However, down-regulated miR-143 significantly enhanced cell viability and the effect could be abrogated by ERK5 or MAPK7 silence. Furthermore, down-regulated miR-143 increased expression of cyclin D1 and the increase was abrogated by ERK5 silence. The increase of cell viability induced by miR-143 down-regulation could be reversed by cyclin D1 silence.

The functional role of miR-143 in tumor had been studied in recent years. Our study was consistent with a previous study that reported that miR-143 level was down-regulated in BC (12). Increasing evidence has demonstrated the potential role of miR-143 in cell proliferation in cancers (22). For example, overexpression of miR-143 was reported to suppress cell proliferation through regulating EGFR/RAS/MAPK pathway in prostate cancer cells (11). The study of Chen et al. (23) suggested that up-regulated miR-143 significantly inhibited colorectal cancer cell growth through targeting KRAS. Liu et al. (24) reported that overexpression of miR-143 inhibited the formation of tumors *in vivo* and human cervical cancer cell proliferation *in vitro* by regulation of Bcl-2. A study of bladder carcinoma also demonstrated an inhibitory effect of miR-143 on cell proliferation by targeting cyclooxygenase-2 (25). Similarly, our study also revealed that up-regulated miR-143 remarkably inhibited cell growth. Our results indicated the anti-proliferation role of miR-143 in BC.

To further investigate the mechanism of anti-proliferation role of miR-143, we detected the expression of ERK5. ERK5 is a member of the MAPK family, which has been reported as an enhancer of cell proliferation and progression by mediating the cell cycle, as well as a tumorigenesis (26). A previous study had suggested that miR-143 could influence the MAPK pathways, key for oncogenesis, by acting on ERK5 in prostate cancer (27). Charni et al. (28) reported that the down-regulated ERK5 could effectively reduce leukemia cell viability. Zhai et al. (29) also reported that miR-143 could inhibit tumor growth of BC through down-regulation of ERK5. Thus, we assumed that there might be an association between miR-143 and ERK5 in BC. Consistently, our study demonstrated a negative relationship between miR-143 and ERK5 in both BC tissues and cells. Moreover, our study confirmed that silencing of ERK5 significantly reduced cell viability. When ERK5 expression was inhibited, suppression of miR-143 could not influence cell viability, indicating that the anti-proliferation role of miR-143 might be associated with ERK5 expressions in MCF-7 cells.

In addition, we also explored the alteration of MAP3K7 expression. MAP3K7, a serine/threonine kinase of the MAP3K family, is known as a TGF-β-activated kinase-1 and can be quickly stimulated by TGF-β signal transduction (30). MAP3K7 has been considered to be an important regulator of many cellular pathways associated with cancer cell growth. Down-regulated MAP3K7 has been reported to promote cancer cell death in BC (31). Also, suppression of MAP3K7 signaling could inhibit the growth of human head and neck squamous cell carcinoma and BC cells (32,33). In line with the above studies, our results corroborated that MAP3K7 levels were elevated in BC tissues, and knockdown of MAP3K7 significantly inhibited cell growth in MCF-7 cells. Furthermore, our study also found that up-regulated miR-143 inhibited the level of MAP3K7 and the cell viability was not significantly altered by simultaneous suppression of miR-143 and MAP3K7, indicating that the anti-proliferation role of miR-143 might be at least partly controlled by regulation of MAP3K7 expressions in MCF-7 cells. However, some studies reported that the deletion of MAP3K7 gene promoted cell proliferation, migration, and invasion in high-grade prostate cancer (34), as well as induced liver cancer (35), suggesting the tumor suppressor role of MAP3K7. This contradiction might be caused by different cell types used in the studies or carcinoma progression.

Cyclin D1 is a key regulator of the cell progression, essential for the G1 phase (36). Increased expression of cyclin D1 is an early event in cancer cells and cyclin D1 is regarded as an oncogene (37). A previous study reported that miR-143 inhibits cyclin D1 expression in prostate cancer cell lines (38). Hence, we hypothesized that miR-143 might regulate the expression of cyclin D1 in BC. We confirmed that overexpression of miR-143 dramatically decreased the levels of cyclin D1 while suppression of miR-143 elevated the cyclin D1 expression. Further results displayed that simultaneous suppression of miR-143 and cyclin D1 just reversed the effects of miR-143 inhibitor on cell viability, implying that miR-143 might affect the cell proliferation of BC cells by negatively mediating the expression of cyclin D1. Moreover, we also revealed that miR-143 regulated cyclin D1 expression through down-regulation of ERK5. Liu et al. found that miR-143 decreased cell proliferation of HepG2 cells due to a G0/G1 arrest of cell cycle (39). Meanwhile, another study also reported that overexpression of miR-143 inhibits cell proliferation through blocking of the G1/S phase transition in human esophageal squamous cell carcinoma. Ectopic expression of miR-143 led to an increased percentage of cells in the G0/G1 phase and decreased percentage of cells in the S or G2/M phase (40). Thus, we hypothesized that miR-143 mimic might decrease cellular proliferation through cell cycle arrest at G0/G1 phase in BC cells.

In conclusion, the present study reveals that both ERK5 and MAP3K7 may be the target genes of miR-143. Increased expression of miR-143 can inhibit cell growth, which may be associated with ERK5 and MAP3K7 expressions in BC.
